# Proton-Mediated PIEZO2 Channelopathy: Linking Oxaliplatin Treatment to Impaired Proprioception and Cognitive Deficits

**DOI:** 10.3390/cancers16233898

**Published:** 2024-11-21

**Authors:** Balázs Sonkodi

**Affiliations:** 1 Department of Health Sciences and Sport Medicine, Hungarian University of Sports Science, 1123 Budapest, Hungary; bsonkodi@gmail.com; 2 Department of Sports Medicine, Semmelweis University, 1122 Budapest, Hungary

**Keywords:** oxaliplatin, PIEZO2 ion channel, proprioception, hippocampus, chemobrain

## Abstract

Oxaliplatin induces acute neuropathy within a few hours post-treatment, with symptoms persisting for several days. Delayed onset muscle soreness also causes the delayed onset of mechanical pain sensation starting at about 6–8 h and lasting up to a week after exercise. Both conditions come with impaired proprioception and could be chronic if these bouts are repeated frequently. The involvement of PIEZO2 ion channels, as the principal mechanosensory channels responsible for proprioception, is theorized in both conditions as well. The current opinion manuscript is meant to explain how the minor stretch-related microdamage of PIEZO2 on Type Ia proprioceptive terminals could explain the aforementioned symptoms of impaired proprioception. This includes a platinum-induced proton affinity ‘switch’ on these proprioceptive endings with PIEZO2 content, resulting in this being the likely initiating cause. Furthermore, it postulates how the proton-based ultrafast long-range oscillatory synchronization to the hippocampus could be impaired due to this microdamage on Type Ia proprioceptive terminals. Finally, the manuscript provides insight into how the impairment of the PIEZO2-initiated ultrafast muscle–brain axis may contribute to chemobrain and its associated cognitive and memory deficits.

## Simple Summary

This opinion paper postulates how the proton-based ultrafast long-range oscillatory synchronization to the hippocampus could be impaired due to oxaliplatin-induced microdamage on Type Ia proprioceptive terminals. Accordingly, PIEZO2 ion channels on these proprioceptive endings may endure a proton affinity ‘switch’ due to oxaliplatin treatment, resulting in an acquired PIEZO2 channelopathy. Finally, this manuscript provides insight into how the impairment of the PIEZO2-initiated ultrafast muscle–brain axis may contribute to chemobrain and its associated cognitive and memory deficits.

## 1. Introduction

Delayed onset muscle soreness (DOMS) is often induced as an intentional exercise technique to achieve muscle growth under the credo of “no pain, no gain”. This delayed, mysterious pain condition is transient, and the muscle gain response is a controlled mechanism. In contrast, cancer is an uncontrolled chronic growth mechanism. Oxaliplatin, platinum-based chemotherapy, is meant to fight against tumor growth by primarily targeting the uncontrolled tumor growth territories.

An apparent analogy in the mechanism of oxaliplatin-induced neurotoxicity, a structural and/or functional impairment of the nervous system, and DOMS is developing acute neuropathy or nerve damage. Acute neuropathy develops a couple of hours after the administration of this third-generation platinum derivative and lasts for days. Correspondingly, the delayed onset of pain sensation evolves a couple of hours after DOMS-inducing exercise and lasts for up to 7 days. It is important to note that the involvement of PIEZO2 in oxaliplatin-induced endothelium-dependent pain has been demonstrated experimentally [1,2]. However, both conditions could be induced by repeated bouts. In addition, oxaliplatin neurotoxicity could become chronic, as DOMS could evolve into a chronic condition as well in the absence of the proper periodization of regeneration. This mechanistic link between oxaliplatin-induced neurotoxicity, DOMS, and neurodegenerative diseases was extrapolated earlier [2].

One distinctive side effect of oxaliplatin, amongst others, is impaired proprioception, as shown experimentally [3]. Impaired proprioception is experienced in DOMS too [4]. Significant findings are that PIEZO2 is the principal mechanosensory ion channel responsible for proprioception [5]. Indeed, it is shown that oxaliplatin is capable of impairing proprioceptive Type Ia fibers in the muscle spindle without causing observable degenerative abnormality [3]. In line with this type of functional deficit, it is theorized that DOMS is initiated by the primary damage, or the PIEZO2 channelopathy of the same intrafusal proprioceptive terminals, in an acquired and autonomous way [6]. Accordingly, PIEZO2 channelopathy relates to the loss of the proper inactivation capability of this ion channel under allostatic stress; hence, it becomes “leaky” when it should not be [6]. Part of this theory that vesicular glutamate release experiences an impairment is that it is meant to sustain the static phase firing encoding during a prolonged stretch on Type Ia proprioceptive terminals [7]. Interestingly, the oxaliplatin-treated proprioceptive nerve exhibits normal excitability experimentally but not under stretching conditions [3]. Correspondingly, the only minor impairment is diminished excitability under prolonged stretching [3]. This alteration is translated as the ‘switch’ of the impaired monosynaptic Type Ia fiber-derived static phase firing sensory encoding to Type II fiber-transduced polysynaptic encoding [2]. This ‘switch’ is likely represented in the significant delay of the medium latency response of the stretch reflex in the primary damage phase of DOMS [7]. Bullinger et al. devoted the cessation of firing under static stretching to mechanically gated sodium channels [3], but later, it was suggested that another mechanosensitive ion channel, namely PIEZO2, should be responsible for this minor alteration [6,7]. In support of this, Na_v_1.1 takes over part of proprioceptive signaling once PIEZO2 is inactivated [7,8] or when PIEZO2 is likely microdamaged [7]. ASIC2 may also contribute to proton-signaled proprioception from Type Ia primary afferent terminals in conjunction with PIEZO2 as well [7,9]. Consequently, the current author proposes that the loss of microdamaged PIEZO2 function may initiate not only aberrant mechanically gated sodium channel functions but impair proton-based ASIC2 signaling on proprioceptive primary afferents [7].

Accordingly, the current opinion paper examines how oxaliplatin may initiate an acquired PIEZO2 channelopathy primarily on proprioceptive terminals of the muscle spindle. Underpinning this microinjury, it is important to note that mechanotransduction system activation has its hierarchical order [10], and PIEZO ion channels, specifically PIEZO2, are likely the first in this hierarchical order of mechanosensitive ion channel activation due to their burst-activating feature and homeostatic regulating capacity [11]. Furthermore, PIEZO2 channelopathy has its longitudinal dimension induced by the repeated bout effect in a dose-dependent manner, and this is depicted first by the quad-phasic non-contact injury model, which, like oxaliplatin treatment, carries these features as well [12].

## 2. The Proton Affinity ‘Switch’

PIEZO2 is a giant stretch-gated, excitatory mechanosensitive transmembrane ion channel. Recently, it was demonstrated that its voltage-block function regulates mechanical pain sensitivity [13]. Even earlier, it was theorized that PIEZO2 is not only a voltage rectifier but may behave like a semiconductor Schottky barrier diode [7]. Furthermore, this theory entails that PIEZO2 may initiate a proton-based ultrafast long-range oscillatory synchronization to the hippocampal theta rhythm from the muscle spindle’s Type Ia primary afferent terminals [7]. The implicit prerequisite of this suggested novel, unaccounted ultrafast long-range neurotransmission is the proper availability of protons [7].

Protonation is modulated by proton affinity, and platinum has not only a high proton affinity but is the prominent material to induce the hydrogen evolution reaction, hence further reducing the proton availability in its nanomilieu [14]. This feature of platinum possibly leads to the water dissociation of interfacial water between the cell membrane and the extracellular matrix. Another important consideration is that available protons control the vesicular glutamate transport (VGLUT), and part of the PIEZO2 channelopathy theory is that the vesicular glutamate release is impaired [7]. Glutamate not only has high proton affinity, but in association with changing transmembrane hydration, it could even alter the protonation of the protein residue by mediating transmembrane proton transfer [15]. Therefore, it is likely that oxaliplatin reduces protonation in association with impaired vesicular glutamate release on primary proprioceptive afferents, leading to the earlier suggested glutamate spillover [2,6]. Indeed, it has been experimentally demonstrated that atypical glutamate receptors on these primary afferent terminals with vesicular glutamate release control stretch sensitivity [16]. Furthermore, protons, in fact, control VGLUT proteins, which are proton- and voltage-activated channels, through allosteric transmission regulation at a distance [17].

It is notable that this proton affinity ‘switch’ is shown in an autonomous light-driven proton pump of bacteriorhodopsin [18]. Accordingly, even a small change in the proton affinity of this protein, leading to a non-equilibrium state, could be sufficient to stem unidirectional transmembrane proton transport in the absence of an accessibility path for protons [18]. The current author suggests that oxaliplatin could induce such a proton affinity “switch” on the protonation of PIEZO2 channels with resultant unidirectional transmembrane proton transport and the impaired cargo loading of vesicular glutamate release in the absence of proton availability, leading to glutamate spillover. This is not to mention that the proposed autonomously acquired microdamage on PIEZO2 channels in DOMS may also cause such a conformational change that alters the protonation of the protein structure through the proton affinity ‘switch’ and the unidirectional transmembrane proton transport due to the dissociation of auxiliary inhibitor ligands of PIEZO2 [7], leading to the earlier suggested ‘leakiness’ of PIEZO2 [6].

The aforementioned ‘switch’ is analogous to the impairment of the semiconductor Schottky barrier diode-like feature of PIEZO2 on the Type Ia terminals in association with the impairment of the vesicular glutamate signaling, resulting in a ‘switch’ to glutamate-based neurotransmission [7]. Hence, this ‘switch’ is suggested to impair the PIEZO2-initiated proton-based ultrafast proprioceptive feedback to motoneurons and long-range synchronization to the hippocampal theta rhythm [7]. It is indicative that Bullinger et al. observed that they could restitute this deficient sensory transduction in oxaliplatin-treated rats by vibration, and they found no impairment in underlying transient potentials [3]. Correspondingly, it should be considered that available protons are necessary for excitatory PIEZO2 activation through proton resonance, and vibration could restitute the burst activation of the vibration detector PIEZO2 ion channels.

To summarize, oxaliplatin may not only decrease proton availability but also reduce the proton affinity of PIEZO2, especially at the intrafusal proprioceptive terminal of Type Ia fibers. Hence, it evolves into neurotoxic microdamage reflected in a unidirectional transmembrane proton transport ‘switch’ and resultant ‘leakiness’ or the so-called acquired PIEZO2 channelopathy.

## 3. Proprioception, Hippocampus, Neurogenesis

**Figure 1:**
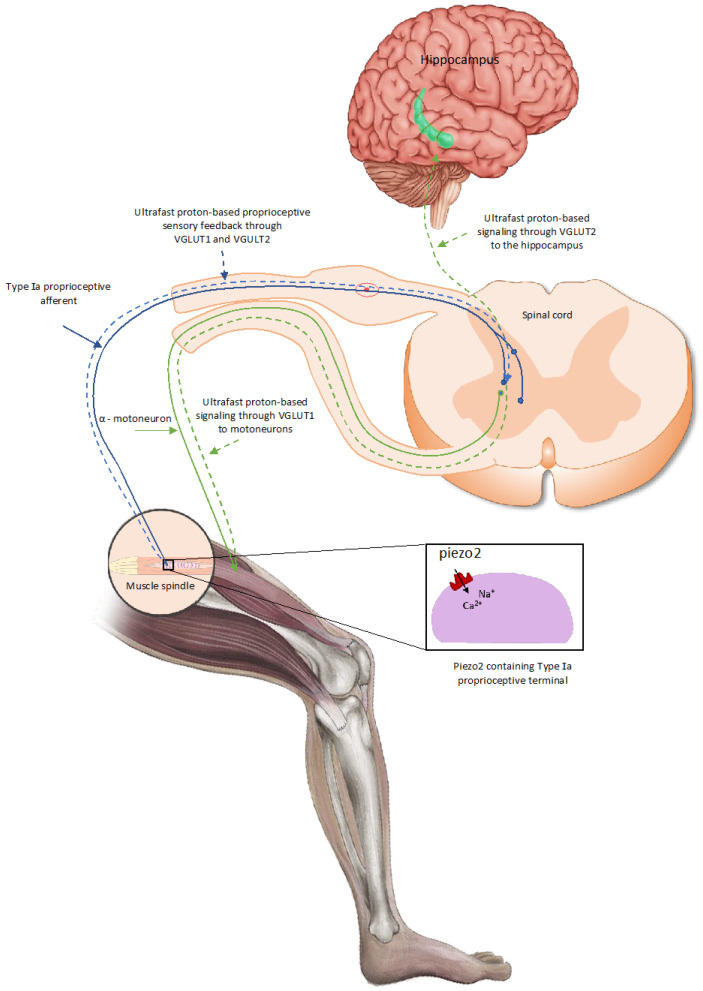
PIEZO2-driven, proton-based ultrafast signaling from intrafusal proprioceptive terminals, and how it links hippocampal theta rhythm via VGLUT2 and motoneuron activity via VGLUT1 [20].

This PIEZO2 microdamage-induced ‘switch’, later also coined as the ”miswiring” [8] of the monosynaptic Type Ia fiber encoded static phase firing encoding to the polysynaptic Type II fiber, which is likely to lead to proprioceptive sensory mismatch at neuromuscular junctions of motoneurons, reflected in the observed impaired proprioception [2]. This proprioceptive sensory mismatch wears out the affected neuromuscular junctions of motor neurons on the chronic path, resulting in the accelerated aging of these junctions [7]. Proprioception is the subconscious sensing of our extremities but may contribute to motor learning and memory by providing ultrafast proton-based proprioceptive feedback to motoneurons through VGLUT1 and spatial/speed inputs to the hippocampal theta rhythm via VGLUT2 [7] (Figure 1).

Research is emerging that the hippocampus has an essential role not only in long-term memory but in short-term memory as well [19]. One telling impairment of hippocampal short-term memory is reflected in the so-called “swap error”, the swapping of the location of seen items [19]. This “swap-error” might be associated with the proposed ‘switch’ or the impaired ultrafast PIEZO2-initiated proton-based long-range muscle–brain signaling of spatial/speed inputs through VGLUT2 [7]. The current author suggests that this proposed ‘switch’ contributes to ‘chemobrain’: a phenomenon often reported by cancer survivors to express the thinking and memory problems that are experienced during and after cancer treatment.

The hippocampus is also a site for adult neurogenesis. Another hallmark finding of Bullinger et al. is the decreased platelet count [3]. However, there is no correlation between thrombocytopenia and neurologic complications from oxaliplatin treatment in the scientific literature, despite the fact that the current author would like to propose it. In support, exercise activates platelets, which is certainly PIEZO crosstalk-dependent through PIEZO1 activation, and, in return, this contributes to exercise-driven enhanced hippocampal precursor cell proliferation even in aged mice [21]. Moreover, the increased systemic administration of one platelet-derived exerkine, namely CXCL 4/platelet factor 4, is capable of dampening the age-associated regenerative and cognitive impairment in a hippocampal neurogenesis-related fashion [21]. The current author speculatively suggests that oxaliplatin-induced proprioceptive terminal PIEZO2 channelopathy could impair the aforementioned ultrafast proton-based muscle–brain axis or the bidirectional crosstalk between the muscle and brain in order to maintain the energy homeostasis of the body. Moreover, PIEZO2 channelopathy impairs PIEZO2-PIEZO1 crosstalk within a compartmental micromilieu, and astrocytes contain PIEZO1 [7] even in the hippocampus. It is important to note that astrocytic PIEZO1 determines neurogenesis and cognitive functions through mechanotransduction, and the deletion of PIEZO1 drastically impairs neurogenesis [22]. Hence, one consequence of the PIEZO2 channelopathy-induced impaired muscle–brain axis is the detriment of hippocampal adult neurogenesis, which, in return, contributes to decreased platelet counts in the long run. Hence, the crosstalk between platelets and hippocampal neurogenesis may exist bi-directionally, or more precisely, there might be an underlying exercise-dependent like adult hippocampal neurogenesis modulation on platelet count, but this is suggested to be impaired in the presence of PIEZO2 channelopathy. One further indirect indication of this possible link between platelet regulation and the nervous system is that the neurodegeneration skew platelet may function beyond hemostasis by inducing brain function-derived alternative platelet activation pathways [23].

The current author further suggests that oxaliplatin contributes to the upregulation of PIEZO1 channels of liver sinusoidal endothelial cells in a feedforward manner due to PIEZO2 channeloapthy-induced impaired PIEZO2-PIEZO1 crosstalk. In support, PIEZO2 channelopathy-derived impaired PIEZO2-PIEZO1 crosstalk downregulates PIEZO1 on certain cells, like dendritic cells, and upregulates PIEZO1 on other cells, like satellite/astrocyte cells within the affected compartments or organs with blood barriers [7]. PIEZO1 channels are indeed present in liver sinusoidal endothelial cells with the role of shear stress detection [24]. The oxaliplatin-derived PIEZO2 channelopathy induces impaired PIEZO1-PIEZO2 crosstalk in the short-term and PIEZO1 upregulation in the long-term on these liver sinusoidal endothelial cells. For the short term, the sinusoidal endothelial cells are disrupted [25], likely due to the impairment of PIEZO2-PIEZO1 crosstalk. Moreover, this disruption allows platelet extravasation and activation [25]. The higher the hepatic sinusoidal injury, the higher the increase in spleen size, leading to thrombocytopenia [26]. PIEZO1 exerts a brake on megakaryocyte maturation and platelet formation, and the higher the PIEZO1 expression on bone marrow megakaryocytes, the lower the platelet count [27].

However, oxaliplatin-induced thrombocytopenia does not affect the megakaryocyte number in the bone marrow [28], and the sensitivity to oxaliplatin is likely due to PIEZO2 channelopathy. In support, complete loss-of-function mutations in PIEZO2 result not only in loss of pain but loss of sensitization as well [29]. However, the acute form of PIEZO2 channelopathy rather resembles gain-of-function mutation due to its aforementioned “leakiness” when it should not be; hence, it enhances sensitivity. Furthermore, PIEZO2 channelopathy is also suggested to induce the so-called gateway reflex [7], a neuro-immune interaction for the regulation of regional vessels [30], and even tissue-specific autoimmune mechanisms [31]. The clinical cases of immune-mediated acute thrombocytopenia resulting from sensitivity to oxaliplatin [28] could be the result of the PIEZO2 channelopathy-induced gateway reflex.

It should not be excluded that oxaliplatin may cause a proton affinity ‘switch’ on the PIEZO1 of bone marrow megakaryocytes, further contributing to thrombocytopenia. An interesting recent indirect supportive finding is that the deletion of nine amino acids causes a PIEZO1 variant of unknown significance, which may cause higher erythrocyte count dysregulation acutely due to an over-excessive exercise regimen, including erythrocyte count drop [11]. Five out of nine amino acid deletions were glutamate, and three were glutamine [11]. Both of these amino acids have high proton affinity; therefore, their loss-derived proton affinity decrease in PIEZO1 might have a functional role in the acute dysregulated erythrocytes count, including the erythrocyte count drop.

Briefly, this opinion paper posits that the oxaliplatin-induced proton affinity ‘switch’ on proprioceptive terminal PIEZO2 impairs the ultrafast proton-based long-range neurotransmission to the hippocampus through VGLUT2, leading to hippocampal memory deficit on the short and long-term as well. This impairment of oscillatory proton signaling through the muscle–brain axis may not only contribute to ‘chemobrain’, but to the dysregulated activation of adult neurogenesis, alternative platelet activation pathways, and immune-mediated acute thrombocytopenia.

## 4. Pain and the Hippocampal Link

Last but not least, the hippocampus is also a critical locus in both acute and chronic pain modulation and formation, respectively. Therefore, it likely contributes to acute or chronic neuropathic pain from oxaliplatin treatment due to the ‘switch’ to glutamate-based neurotransmission. The fact that the voltage-block function of PIEZO2 regulates mechanical pain sensitivity is a major step forward in pain science [13]. It could underline an earlier theory that the autonomously acquired channelopathy of PIEZO2 is the autonomous pain generator that drives central sensitization in an acute transient manner in DOMS [6] and in chronic conditions as well. After all, it seems that the biophysical disintegrative ‘switch’ is needed for mechanical pain sensitivity regulation as part of the impaired Schottky barrier diode function of PIEZO2. This primary ‘switch’ is a transformation from proton handling for quantum tunneling and semiconduction for transmembrane proton transfer, and that is the equivalent ‘switch’ to glutamate signaling from a vesicular glutamate-based one. Wide dynamic range neurons are suggested to play a central regulatory role on the spinal dorsal horn in this mechanical pain sensitivity regulation as the gate controllers of pain [7]. Accordingly, as long as only the non-painful stimuli of PIEZO2 channelopathy on Type Ia terminals prevail, then no pain is associated with the primary damage phase [2,6]. However, when the painful stimuli evolve on C-fibers due to PIEZO2 channelopathy on Type III fibers in the secondary damage phase of DOMS, then the painful stimuli could prevail [7]. This painful stimulus is likely modulated by the aforementioned endothelium-dependent manner, as was observed in oxaliplatin therapy [1]. In support of this, recent research demonstrated that Piezo2 may induce an intrinsic oscillatory interoceptive mechanism through a pressure pulsation transduction pathway that modulates olfactory bulb activity in arousal and it is synchronized to brain activities [32], as was suggested earlier [33]. Hence, the current author proposes in mechanical hyperalgesia that the PIEZO2 content of C-fibers is modulated by endothelial PIEZO2 through a PIEZO2-PIEZO2 low-frequency crosstalk that is based on the intrinsic oscillatory interoceptive nature of this ion channel. This not only modulates neuropathic mechanical pain but impacts brain oscillations and synchrony at different frequencies, including at low frequencies, due to the low-frequency noise of the semiconductor Schottky barrier diode-like feature of PIEZO2.

Furthermore, exercise could turn back the PIEZO2 ‘switch’ by gating to non-painful stimuli on WDR neurons in DOMS because it activates the gating of otherwise functionally microdamaged proprioceptive PIEZO2 channels, hence causing exercise-induced analgesia. With the termination of exercise, mechanical pain returns since the microdamaged, deactivated proprioceptive terminal PIEZO2 are leaky and, therefore, cannot place a block on the painful stimuli anymore. It is notable that vibration through the activation of oxaliplatin-microdamaged PIEZO2 could mimic exercise stimulus as experimentally presented by Bullinger et al., hence causing exercise/vibration-induced analgesia. However, on the chronic path, neuropathic pain could evolve with hippocampal involvement as a consequence of oxaliplatin treatment.

To put it concisely, the suggested platinum-induced proton affinity ‘switch’ on the PIEZO2 function of Type Ia proprioceptive endings impairs the ultrafast proton-based long-range oscillatory synchronization to the hippocampus, resulting instead in glutamate-based signaling and pain inducement with hippocampal modulation.

## 5. Conclusions and Future Directions

In summary, oxaliplatin may shed light on how the biophysical fragmentation of the two critical functions of PIEZO2 could result in the gateway to pathophysiology as the primary damage or root cause of aging. Therefore, it is not only the gating of PIEZO2 and voltage block counts that is important but the availability of protons for the protonation of PIEZO2 in order for proper semiconductor Schottky barrier diode-like channel functions. In the absence of sufficient protonation due to conformational changes in the protein structure, this may induce a proton affinity ‘switch’ with unidirectional transmembrane transport that decapacitates this semiconductor Schottky barrier diode-like feature of PIEZO2 and leads to the proposed oxaliplatin-induced PIEZO2 channelopathy or PIEZO2 leakiness [6]. It is noteworthy that a functionally analogous autonomously acquired PIEZO2 channelopathy is proposed in DOMS and amyotrophic lateral sclerosis as well due to the dissociation of the auxiliary connection of the inhibitor protein ligands of PIEZO2, like MyoD [7] or TMEM120A [34]. The presence of PIEZO2 on proprioceptive Type Ia terminals has special relevance due to the suggested PIEZO2-initiated proton-signaled ultrafast long-range synchronization to hippocampal theta rhythm [7]. This novel, unaccounted proton affinity ‘switch’ mechanism on PIEZO2, coined as acquired PIEZO2 channelopathy [7], is suggested to be the cause of why chronic systemic oxaliplatin treatment will result not only in large fiber neuropathy but in diminished hippocampal theta activity and adult neurogenesis, not to mention it could disclose the enigmatic learning and memory deficits leading to ‘chemobrain’ [35]. This ‘switch’ to PIEZO2 channelopthy has been suggested as the primary damage or one principle gateway to pathophysiology [7]. Keeping the ‘switch’ turned on chronically will lead to accelerated aging, as could be observed on impaired proprioceptive Type Ia fibers that eventually wear out the neuromuscular junctions of affected motoneurons [7], in addition to potentially leading to ‘chemobrain’.

One suggested way of unloading the ‘switch’, or the unloading of undesired mitochondrial energy-generating pathways, is exercise without overloading the proprioceptive terminals of the muscle spindle [2]. The emergence of cancer-targeting exercise therapy has gone through a remarkable transition lately from a “does not hurt” standpoint to the realization that exercise can exert direct control on tumor biology by affecting tumor intrinsic factors. Accordingly, exercise intensity, duration, frequency, and mode of exercise [36] should be prescribed if we consider exercise therapy to be a form of ‘medication’. One important consideration is the Warburg-type highly glycolytic metabolism aspect of tumors [36]. In vitro experimental studies show that even one single bout of exercise suppresses growth in certain cancer cell lines, but further acute bouts add up in the “dose” of this ‘medication’ [37]. Even high-intensity anaerobic exercise has been demonstrated to be safe and effective by exhibiting stronger tumor growth reduction through inhibiting glycolysis pathways [36].

However, the current author suggests that the metabolic aspect of tumor and proprioceptive terminal microenvironments should be considered simultaneously under oxaliplatin-induced neurotoxicity. Correspondingly, not only glycolysis but glutaminolysis should be contemplated when it comes to tumor growth [38] and proprioceptive terminal micromilieus. In particular, glutamine fermentation [38] may posit a competitive advantage to tumor metabolism regardless of its constraint on tumor growth because this is when the astrocyte-neuron lactate shuttle-like mechanism is suggested to be derailed in association with PIEZO2 channelopathy-induced impaired glutamate vesicular release at proprioceptive terminals [7]. Therefore, high-intensity anaerobic eccentric or resistance exercise is counter-advised due to the likely glutamine fermentation pathway promotion, which is clearly further damaging to proprioceptive terminals under oxaliplatin-induced neurotoxicity.

Exercise has a positive effect on fatigue, quality of life, and the physical functioning of oxaliplatin-treated patients [39]. Nonetheless, eccentric exercise, especially fatiguing ones, should be avoided, and concentric exercise should be promoted, in line with home-based aerobic exercise findings [40], but with minimal position sense loading [41] under oxaliplatin treatment. Stationary cycling with minimized resistance and for up to 10 min would be such a regimen, preferably at the end of the day [41]. This exercise protocol is beneficial for cancer patients as well. An important consideration is that PIEZO2 is likely inactivated at around 10 min when the low-frequency power of heart rate variability drops to almost zero [33], and thereafter, exercise loading under “leaky” inactivated PIEZO2 is recommended to be avoided by oxaliplatin-treated patients.

Glutamine supplementation [42] is another promising future direction due to the aforementioned metabolic challenge; however, patient-specific routes, doses, and forms of administration require experienced expertise [43], and further clinical studies are still needed from this perspective [44]. An indicative finding is that L-glutamine supplementation attenuates exercise-induced muscle damage under eccentric exercise in athletes [45], as this type of damage is part of the proprioceptive PIEZO2 channelopathy theory of DOMS [7]. Cancer patients under oxaliplatin treatment may benefit from L-glutamine supplementation due to their compromised energy generation mitochondrial pathways not only in the cancer microenvironment but, more importantly, in proprioceptive terminal micromilieu, but this likely beneficial impact may come only with aerobic exercise loading for up to 10 min. The combination of stationary cycling for up to 10 min with minimized resistance and glutamine supplementation might be an interesting future direction in order to unload the undesired ‘switched’ mitochondrial energy-generating pathways in cancer treatment and oxaliplatin-induced neurotoxicity. The good news is that excellent animal models are available for oxaliplatin-induced proprioceptive impairment [3] for future research. Accordingly, human trials meant to assess the safety and efficacy of this recommended combined treatment effort on oxaliplatin-treated cancer patients must be preceded by well-designed animal studies. The author calls for this cautious approach because it should not be excluded that glutamine supplementation may impair the efficacy of oxaliplatin treatment.

The author would like to emphasize the limitation of this opinion paper in that no unquestionable direct electrophysiological evidence of acquired PIEZO2 channelopathy exists. However, indirect findings of acquired PIEZO2 microdamage are on the rise at an unprecedented rate; we thank Nobel Laureate Ardem Patapoutian for his discovery of PIEZO ion channels. Acquired PIEZO2 channelopathy was first suggested in 2021 [6], and for years, the scientific community has been skeptical about the feasibility of this acquired microdamage, but recently, more and more scientists have confirmed this feasibility [46,47].
